# Current Review of Mycotoxin Biodegradation and Bioadsorption: Microorganisms, Mechanisms, and Main Important Applications

**DOI:** 10.3390/toxins14110729

**Published:** 2022-10-25

**Authors:** Seyni Ndiaye, Minhui Zhang, Mouhamed Fall, Nicolas M. Ayessou, Qi Zhang, Peiwu Li

**Affiliations:** 1 Key Laboratory of Biology and Genetic Improvement of Oil Crops, Ministry of Agriculture and Rural Affairs, Wuhan 430062, China; 2 Oil Crops Research Institute, Chinese Academy of Agricultural Sciences, Wuhan 430062, China; 3 Key Laboratory of Detection for Mycotoxins, Ministry of Agriculture and Rural Affairs, Wuhan 430062, China; 4 Laboratory of Risk Assessment for Oilseeds Products (Wuhan), Ministry of Agriculture and Rural Affairs, Wuhan 430062, China; 5 Laboratoire D’Analyses et D’Essai, Ecole Supérieure Polytechnique, Université Cheikh Anta Diop, Fann-Dakar 5085, Senegal; 6 Key Laboratory of Agro-Products Processing, Institute of Agro-Products Processing Science and Technology, Chinese Academy of Agricultural Sciences, Ministry of Agriculture, Beijing 100193, China; 7 Hubei Hongshan Laboratory, Wuhan 430070, China

**Keywords:** mycotoxins, aflatoxins, contamination, microorganisms, biodegradation, enzymes

## Abstract

Mycotoxins are secondary metabolites produced by fungi. Food/feed contamination by mycotoxins is a great threat to food safety. The contamination can occur along the food chain and can cause many diseases in humans and animals, and it also can cause economic losses. Many detoxification methods, including physical, chemical, and biological techniques, have been established to eliminate mycotoxins in food/feed. The biological method, with mycotoxin detoxification by microorganisms, is reliable, efficient, less costly, and easy to use compared with physical and chemical ones. However, it is important to discover the metabolite’s toxicity resulting from mycotoxin biodegradation. These compounds can be less or more toxic than the parent. On the other hand, mechanisms involved in a mycotoxin’s biological control remain still unclear. Mostly, there is little information about the method used by microorganisms to control mycotoxins. Therefore, this article presents an overview of the most toxic mycotoxins and the different microorganisms that have a mycotoxin detoxification ability. At the same time, different screening methods for degradation compound elucidation are given. In addition, the review summarizes mechanisms of mycotoxin biodegradation and gives some applications.

## 1. Introduction

Mycotoxins are secondary metabolites with low molecular weight produced by filamentous fungal species [1,2,3]. Their chemical structures are very different [4], and they cause various degrees of toxicity in humans and animals. Mycotoxins are often genotypically specific but can be produced by one or more fungal species; one species can produce more than one kind of mycotoxin. In the environment, there are more than 200 kinds of mycotoxins [5]. Some of them can exhibit carcinogenic, teratogenic, mutagenic, and neurotoxic properties, and others can show antitumor capacity and cytotoxic and antimicrobial properties [4,6].

Mycotoxin contamination can occur throughout the whole food process, from pre-harvest to food storage [5,7,8,9]. It is estimated that 25% of the world’s agricultural products may be contaminated by mycotoxins each year [10], which leads to economic losses and causes a variety of toxic effects in humans and animals. According to the RASFF (Rapid Alert System for Food and Feed), for the 10-year period from 2010–2019, almost 98.9% of U.S. food notifications on mycotoxins were due to aflatoxin contamination in almonds, peanuts, and pistachio nuts [11]. A multi-mycotoxin analysis of sorghum and finger millet in 2014 showed that these two types of cereals were contaminated with major mycotoxins, with a prevalence of 6 to 52% for finger millet and less than 15% for sorghum [12]. A similar study about the occurrence of mycotoxins in peanuts and peanut products showed that the level of aflatoxins was higher than the maximum limit in 90% of the samples [13]. A study by Monyo et al. on the occurrence of aflatoxin contamination in groundnut demonstrated that the amount of aflatoxin was higher than the maximal limit in 11 to 28% of the samples and below the limit in 2 to 10% of the samples [14]. A study on the occurrence of ochratoxin A (OTA) in food products available in Silesia markets showed that around 22% of the samples were contaminated [15]. Up to 30 or 31% of total wheat-based product samples collected from some districts of Punjab were found to be contaminated with aflatoxins and zearalenone (ZEN) [16]. A three-year survey about Fusarium mycotoxin contamination in wheat samples showed the presence of deoxynivalenol (DON) and nivalenol (NIV) in about 540 and 337 μg/kg, respectively [17]. To deal with this worldwide problem, many detoxification methods have been found against mycotoxins: physical methods, chemical methods, and biological methods [18,19].

Physical control refers to all methods that use the physical properties of a detoxication agent. This can include adsorption, extrusion, cooking, ozonation, the mechanical separation of the clean product from contaminated one, heating at high temperatures, use of radiation and light, grinding, and washing [20,21]. At present, the utilization of mycotoxin-binding adsorbents is the most frequently applied method to protect animals from contaminated feed [22]. Agro-product processing can also reduce mycotoxin contamination. Fermentation has been useful for some Fusarium mycotoxins [23]. It is considered an excellent technique for mycotoxin control in African countries [24].

Chemical control refers to methods that require the use of chemical compounds. This includes techniques such as ammonization [25], the influence of acids and bases, and the influence of oxidizing agents or various inorganic and organic chemicals [20]. However, these methods have some limitations because of the possible deterioration of animal health caused by excessive residual chemical substances in the feed and even some environmentally negative impacts [22].

Nowadays, the biological control of mycotoxins has gained great interest because most chemical and physical detoxification pathways have limitations such as high cost, residual compounds in food and feed, and loss of nutrients. Biological methods include the action of yeasts, bacteria, and enzymes against mycotoxins [26,27]. This detoxification pathway offers an excellent alternative to eliminate toxins and safeguards the nutritional value of food and feed. Nonetheless, biodegradation can result in more toxic compounds. Therefore, there is a need to study the toxicity of the resulting compounds [28].

This paper first describes the most common mycotoxins, then it provides a summary of different mycotoxin detoxification methods by microorganisms and detoxification mechanisms already found. Finally, some important microorganism applications are provided.

## 2. Major Mycotoxin Overview

Along the food chain, aflatoxins, ochratoxin A, zearalenone, deoxynivalenol, nivalenol, fumonisin B1 and B2, and patulin are the most common mycotoxins that can contaminate food and feed [29].

Aflatoxins are secondary fungi metabolites mostly produced by *Aspergillus flavus*, *Aspergillus parasiticus*, *Aspergillus nominus*, and *Aspergillus niger* [5,30,31]. Approximately 18 aflatoxins have been identified [32], but the most common are aflatoxin B1 (AFB1), aflatoxin B2 (AFB2), aflatoxin G1 (AFG1), aflatoxin G2 (AFG2), aflatoxin M1 (AFM1), and aflatoxin M2 (AFM2). Due to their capacity to bind with the DNA of cells, aflatoxins affect protein synthesis [33]. Group B has blue fluorescence and group G has green fluorescence under ultraviolet light [33]. Aflatoxin contamination occurs mainly in hot and humid regions [34]. AFB1 is the most toxic and is cancerogenic, teratogenic, and mutagenic [35]. It is included in category 1A of active carcinogenic compounds (IARC, 1993). The liver is its number one target [36]. On the other hand, AFM1 is a metabolite of AFB1 mainly present in dairy products [37] and is included in group 2B by International Agency for Research on Cancer (IARC) (1993) with a maximum of 0.5 μg/kg in milk [38]. Aflatoxin B1 is bio-transformed into AFB1-8,9-epoxide via cytochrome p450 enzymes, which can induce DNA damage [39].

Patulin (PAT) is a mycotoxin produced mostly by penicillium [40], Byssochlamys, and Aspergillus species [41]. Patulin contamination can cause a lot of damage to animals, such as cancer, by affecting different organs, including the kidney, liver, and intestine [42]. It can contaminate foodstuffs such as fruits and vegetables, especially apples and apple by-products [43,44,45].

Ochratoxin A (OTA) is the most common toxin in grapes and grape-derived products [46], but it can also contaminate food such as coffee, spices, beer [47], and some meat products [48]. OTA is mainly produced by *Aspergillus ochraceus* and *Penicillium verrucosum* [49]. *Aspergillus carbonarius*, and *Aspergillus niger* can also produce OTA, especially in grapes and wines [50]. OTA is very stable at high temperatures [51]. It has neurotoxicological effects [52,53] nephrotoxic effects and can affect mammary functions [54]. OTA production in grapes and grape-derived products is a severe problem in the wine production field, especially in European countries where the climate conditions favor the growth of ochratoxigenic Aspergillus species. Thus, since March 2002, maximum OTA levels in cereals and dried vine fruits are regulated by the EU [55,56].

Fumonisin B1 (FB1) is the most abundant and toxic of the more than 15 types of fumonisins that have been identified [57]. FB1 can cause many diverse toxic effects in animals, including neurotoxicity, hepatotoxicity, and nephrotoxicity [58]. FB1 is a mycotoxin produced by *Fusarium* species such as *Fusarium verticilloides* and *Fusarium proliferatum* [59]. It is found in various crops, but mostly in corn and corn-based food or feed products. It is classified by the IARC 2002 as a carcinogen to humans (group 2B) [60]. 

Trichothecene mycotoxins are a group of sesquiterpenoid metabolites produced by Fusarium species. They usually contaminate cereals and threaten human and animal health [61]. Around 200 tetracyclic sesquiterpenoids have been identified as part of the trichothecene group [62]. Deoxynivalenol (DON) and nivalenol (NIV), and T-2 Toxin (T-2) are the more significant trichothecenes [63]. Type-B trichothecenes include deoxynivalenol (DON), nivalenol (NIV), and their acetylated derivatives, whereas Type-A includes T-2 and HT-2 toxins [10]. They are distinguished by the presence or absence of a carbonyl group in the C8 position.

Deoxynivalenol (DON) has been found to contaminate cereal crops such as barley [64], wheat [65], and maize, as well as their by-products [66]. It is mainly produced by *Fusarium* species [67]. DON may cause toxic and immune-toxic effects in animal species [6]. It is a potent inhibitor of protein synthesis. Fusarium mycotoxins such as DON and ZEN have been shown to affect liver morphology [68] and to have an immunosuppressive effect [69].

Zearalenone is a β-resorcylic acid lactone [70] that is produced by several species of Fusarium, including *Fusarium graminearum*, *Fusarium culmorum*, *Fusarium cerealis*, *Fusarium equiseti*, and *Fusarium semitectum* [71]. This mycotoxin infects cereals such as maize and wheat and can cause many hazards to humans and animals, such as cytogenetic toxicity, decrease fertility, embryotoxicity, and immunotoxicity [72,73,74]. ZEN has the ability to bind to the estrogen receptors of a cell, making it hazardous to humans and animals [75]. ZEN is mostly bio-transforming in α-ZEN and β-ZEN [76].

**Figure 1:**
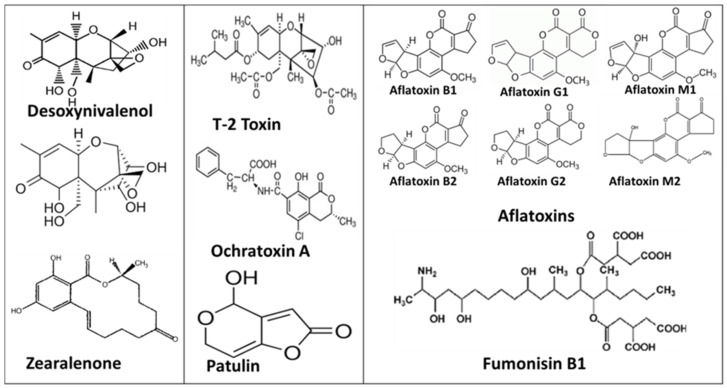
Most common mycotoxin structures.

Due to their toxicity and effects on human health, many countries and international organizations, such as the World Health Organization (WHO), the Food and Agriculture Organization (FAO), and the European Union (EU) through the European Food Safety Authority (EFSA) [77], have set up strict controls of maximum residue levels in foodstuffs. Figure 1 provides some examples of mycotoxin structures.

Table 1 provides an overview of the characteristics of some mycotoxins, including their effects and corresponding recommendations from the World Health Organization [38].

## 3. Microorganism Degradation

### 3.1. Toxin Detoxification by Bacteria

Many species of bacteria have the ability to degrade mycotoxins, including lactic acid bacteria [97] and other species [98]. *Tetragenococcus halophilus* [99], *Rhodococcus erythropolis*, and *Mycobacterium fluoranthenivorans* [100] were proven to degrade AFB1; *Pediococcus parvulus* [101] and *Lactobacillus acidophilus* [102,103] are effective for OTA, AFB1, and AFM1 biocontrol; *Bifidobacterium animalis* [104] is useful for patulin control; *Pseudomonas otitidis* [105] and *Bacillus velezensis* Strain ANSB01E [106] are able to detoxify ZEN. The degradation process depends on many factors, such as the incubation time, the medium, the microorganism species, the concentration of the bacteria cells, and the pH.

The degradation time changes according to the bacteria strain; the microbiota from the thermophilic compost of agricultural waste have degraded AFB1 in 5 days, with a degradation yield of more than 95% after cultivation in a PCS medium at 55 °C [107], and *Rhodococcus pyridinivorans* K408 took 12 days to detoxify AFB1 in bioethanol [26]; the *Lacticaseibacillus rhamnosus* (previously *Lactobacillus rhamnosus)* strains LBGG and LC705, however, removed AFB1 very rapidly [108].

The detoxification rate can depend on the medium; *Bacillus subtilis* UTBSP1 is able to detoxify AFB1 in a higher yield in pistachio nuts than in a medium culture [109], and *Pseudomonas fluorescens* strain 3JW1 can degrade AFB1 in potato dextrose broth and peanut medium by 97.8% and 99.4%, respectively [18].

Many bacteria have been reported to be able to degrade more than one mycotoxin [110]. AFB1 and ZEN have been degraded simultaneously by a microbial consortium, TADC7 [111]; *Rhodococcus pyridinivorans* strains (K408 and AK37) are able to degrade AFB1, T-2, and ZEN simultaneously [22], but also, some *lactic acid bacteria* strains can degrade multi-mycotoxins [112,113]. On the other hand, *Pseudomonas fluorescens* strain 3JW1 is able not only to degrade AFB1 but also to inhibit the AFB1 production of *Aspergillus flavus*. It reduces the amount of AFB1 produced by Aspergillus by 97.8%, 99.4%, and 55.8%, respectively, in the medium culture, peanut medium, and peanut kernels [18]. 

pH also plays an important role in mycotoxin biodegradation. An *Alcaligenes faecalis* strain called ANSA176 is able to detoxify OTA at a rate of 97.43% per 1 mg/mL OTA into OTα within 12 h at 37 °C. The optimal pH is between 6.0–9.0. The bacterial species subjected to the tested pH, ranging from 2.5 to 5.0, were unable to grow [114].

Therefore, mycotoxin biodegradation is an effective method, but it depends on multiple factors. Strict studies are needed for each biocontrol strain to determine the optimal conditions for its use. Table 2 provides an overview of AFB1 detoxification by bacteria with regard to the medium culture used and the main effect on the mycotoxin.

Table 3 provides a global vision of mycotoxin detoxification by bacteria. The main effects on each mycotoxin are provided, as well as the medium culture used.

### 3.2. Mycotoxin Detoxification by Yeast

Yeasts are able to detoxify mycotoxins in different ways: biodegradation, bioadsorption, or the inhibition of mycotoxin production [126].

The biodegradation method can happen with an enzyme isolated from the yeast or the use of the yeast itself. Hong Cao et al. [127] demonstrated the aflatoxin B1 degradation activity of an oxidase enzyme from the fungus *Armillariella tabescens*. The degradation ability of aflatoxin oxidase has been shown by high-performance thin-layer chromatography (HPTLC). The main mechanism was thought to be the cleavage of the bis-furan ring of the aflatoxin molecule. *Meyerozyma guilliermondii* has been shown to be able to control patulin in pear. The patulin degradation ability of *Meyerozyma guilliermondii* in pear wounds increases with a higher concentration of yeast cells. The optimal temperatures are 20 °C and 4 °C in wounds, as well as in whole fruits [128].

On the other hand, yeast biocontrol can involve bioadsorption mechanisms. Some *Saccharomyces* strains are able to remove OTA contamination via adsorption; the mechanism of removal can be enhanced from 45% to 90% by heat treatment of the microorganism and with a lower pH in the medium [129]. In another case, during OTA reduction caused by *Saccharomyces cerevisiae*, the addition of sugar at a temperature of 30 °C enhanced the OTA reduction rate in a semi-synthetic medium [130]. The binding capacity of AFB1, ZEN, OTA, and DON with respect to the *Saccharomyces cerevisiae* contained in beer fermentation residue was studied by Campagnollo et al. [131]. The results showed that beer fermentation residue has a higher binding capacity for ZEN at levels of 75.1% and 77.5% at pH 3.0 and 6.5, respectively. The volatiles of non-fermenting yeasts have shown significant binding activity against mycotoxins. The highest mycotoxin binding activities of these strains were noted against ochratoxin A (92%), AFB2 (66%), AFG2 (59%), and AFB1 (31%) [132]. One issue concerning mycotoxin biocontrol by yeast is that it can sometimes be a reversible mechanism, as has been noted with *S. cerevisiae* CECT 1891 and *L. acidophilus* 24, which were able to remove FB1 from a liquid medium. The removal was a fast and reversible process [133]. Yeasts’ complicated interactions with mycotoxins indicate that cell wall structural integrity, physical structure and morphology, and chemical components all play important roles in the adsorption process. On this basis, future approaches may rely on combinations of different microorganisms to provide complementary advantages in mycotoxin adsorption by yeast [134].

Finally, mycotoxin biocontrol by yeast can concern the inhibition of mycotoxin production. Ponsone et al. studied the activity of some yeast strains isolated from Argentinean vineyards against the growth of the ochratoxigenic *Aspergillus* strain Nigri and also evaluated their effects on OTA. This study demonstrated the natural occurrence of biocontrol agents in the environment to reduce fungi and mycotoxin problems. The results showed that these yeast strains have the ability, under different water activity (aw) and temperature conditions, to control *Aspergillus carbonarius* and *A. niger* aggregate growth and OTA accumulation with a reduction of at least 50% [135]. The same results were obtained when non-fermenting and low-fermenting yeasts were used by Fiori et al. to reduce OTA contamination in grape juice [136]. Nonetheless, some yeast strains are just able to inhibit growth parameters but not mycotoxin production.

Table 4 provides a summary of mycotoxin detoxification by yeast. Emphasis is given to related medium culture and its main effects on mycotoxins.

### 3.3. Toxin Detoxification by Enzymes

Some enzymes isolated from microorganisms or mushrooms are able to degrade one or multiple mycotoxins. This is the case for the Ery4 laccase from *Pleurotus eryngii*, which can degrade AFB1, FB1, OTA, ZEN, and T-2 at the same time [142]. Other enzymes can detoxify only one mycotoxin; this is the case for *Armillariella tabescens*, which has been demonstrated to have an AFB1 degradation ability [127]. The degradation mechanism depends on the enzyme type and the type of mycotoxins. Enzymes can transform the parent into a new compound [91,127,143] or digest it completely [122]. Zeinvand-Lorestani et al. studied the action of a laccase enzyme against AFB1. Under optimal conditions, 67% of the total amount of AFB1 was degraded by the laccase after two days. The degraded product’s prooxidative properties and mutagenicity were lower than the AFB1 one [144]. *Bacillus amyloliquefaciens* ASAG1 can detoxify OTA by 98.5% after 24 h of incubation and 100% after 72 h. On the other hand, the carboxypeptidase cloned from the bacterium is also able to degrade OTA at a level of 41% and 72%, respectively, when cultivated with the supernatant and the purified protein of the carboxypeptidase [145]. Another study showed the effect of carboxypeptidases against OTA. Commercial protease A, commercial pancreatin, and an enzyme extract isolated from *Aspergillus niger* MUM have been proven to degrade OTA to Otα, respectively, by 87.3%, 43.4%, and 99.8% under the optimal conditions of pH 7.5 and temperature 37 °C after 25 h [146]. Porcine pancreatic lipase degraded PAT in pear juice [147].

Table 5 provides an overview of mycotoxin degradation by enzymes with emphasis given to the medium culture and its main effects.

## 4. Detoxification Mechanism

### 4.1. Biodegradation Mechanism

The toxin biodegradation mechanism depends on the microorganism and toxin nature. In their study of AFB1 biodegradation, J. Li et al. demonstrated that aflatoxin B1 degradation by *Tetragenococcus halophilus* is first caused by adsorption and then by the enzymatical pathway. The amount of AFB1 binding caused by adsorption was smaller than the one degraded by the enzymatical pathway. Two mechanisms have been offered as possible pathways for enzymatical action, and six degradation products have been identified: C_14_H_10_O_4_, C_18_H_16_O_8_, C_14_H_12_O_3_, C_16_H_20_O_4_, C_14_H_16_O_2_, and C_14_H_20_O_2_. The first pathway involves the lactone ring, and the second one involves the double bond of the furan ring. Both mechanisms result in the same compound: C_14_H_20_O_2_ [99]. The same results were obtained with another salt-tolerant *Candida versatilis*, CGMCC 3790 [139]. In that case, four resulting compounds were identified by LC/TOF-MS: C_14_H_10_O_4_, C_14_H_12_O_3_, C_13_H_12_O_2_, and C_11_H_10_O_4_. Elsewhere, Hong Cao et al. suggested that the aflatoxin oxidase (AFO) extracted from *Armillariella tabescens* detoxifies the AFB1 by cleaving the bis-furan ring [127]. Adebo et al. found that the pathway of AFB1 degradation by the culture and lysate of a *Pontibacter* species is enzymatical and suggested that when the AFB1 is hydrolyzed, it has probably been transformed into new compounds, which were not identified in that paper [118]. AFB1 has been partially bio-transformed into aflatoxin D1 (AFD1) by deleting a mutant of the bacC gene in *Baccilus subtilis* UTB1. The mechanism was a reduction in the double bond of the lactone ring in the coumarin moiety, followed by the hydrolysis of the ester bond and, finally, the des-carboxylation of the yield to aflatoxin D1 (AFD1); all the processes were catalyzed by the BacC [150]. AFD1, AFD2, and AFD3 have been shown to be degradation compounds of AFB1 detoxification by *Pseudomonas putida*. The mechanism might be lactone [151]. *Phanerochaetesordida YK-624* is able to transform AFB1 into AFB1-8,9-epoxide by, firstly, the oxidation of the manganese protease; thereafter, hydrolysis obtains the final product, AFB1-8,9-dihydrodiol [143]

A yeast enzyme, orotate phosphoribosyltransferase, from *Rhodotorula mucilaginosa* was tested against patulin in apple juice samples and under optimum degradation conditions, which are 0.15 g/L of orotate phosphoribosyltransferase for every 1 mg/L patulin at 25 °C for 18 h; the degradation rate of patulin reached over 80% [148]. During a study of patulin degradation by the yeast *Rhodosporidium paludigenum*, the authors of [138] made the statement that the enzyme(s) responsible for patulin degradation synthesis was enhanced by the presence of patulin. In fact, an assay with protein extracted from cells contaminated by patulin was more active than those with proteins from cells grown without patulin. This difference was attributed to the synthesis of the enzyme. Patulin degradation screening of *Saccharomyces cerevisiae*, tested by M. Li et al., showed that the mechanism was enzymatical and that the PAT-metabolizing enzyme production by the yeast cells is not induced by PAT preincubation [27]. These results were not in accordance with those of Ianiri et al., who concluded in their study that the patulin degradation mechanism by the yeast *Sporobolomyces* sp. IAM 13481 can be induced via pretreatment with the mycotoxin; the pre-incubation with patulin can induce the earlier activation of the gene-encoding proteins of the antioxidant system and the proteins involved in the patulin efflux and patulin degradation [152].

Young et al., in their study, showed that microbial isolate microbiota and pure cultures from chicken intestines have the ability to degrade twelve trichothecenes. The degradation compound identification by MS has suggested that the mechanism includes de-epoxidation and or a diacylation, with the route depending on the presence and position of acyl functionalities [153]. In addition, Gao et al. isolated a bacterium, *Eggerthella* sp. DII-9, which has the ability to degrade some types of trichothecenes, including DON, HT-2, T-2 triol, and T-2 tetraol, into other compounds. T-2 triol was degraded into de-epoxy T-2triol (88.0%), de-epoxy HT-2 (8.6%), and de-epoxy T-2tetraol (2.3%). T-2 tetraol was converted into de-epoxy T-2 tetraol (85.9%), and about 2.3% de-epoxy T-2 triol. HT-2 was transformed into de-epoxy HT-2 (81.4%) and 4.7% de-epoxy T-2 triol. To identify the molecular mechanism, the complete genome of DII-9 was sequenced, but the location of the responsible genes was not found. After the enzymatical study, de-epoxidation was found to be a complex phenomenon [62].

The zearalenone degradation of Bacillus pumilus ES-21 was studied by G. Wang et al. The degradation rate was more than 95.7%, and the degradation compound was identified as 1-(3,5-dihydroxyphenyl)-60-hydroxy-l0-undecen-l00-one. Nonetheless, the compound was not very stable and degraded very rapidly. The mechanism was found to be enzymatical and was thought to be due to esterase activity [91]. on the other hand, during the process of ZEN degradation by Bacillus amyloliquefaciens [122], no resulting compounds were detected. It was concluded that during the biodegradation of Zen by the bacteria’s extracellular enzyme, no ZEN derivatives were produced; in fact, a study of ZEN derivative biodegradation by Bacillus amyloliquefaciens, including α-zearalenol, β-zearalenol, α-zearalanol, and β-Zearalanol, resulted in no metabolites. Koch et al. (2014) studied the ZEN detoxification ability of nine different fungal strains of the genera Rhizopus and Aspergillus, which are known to produce and transform steroids. The results showed that all the strains were able to detoxify ZEN. Biodegradation and adsorption happen simultaneously. Five resulting compounds were identified: ZEN-14-sulfate, ZEN-O-14, ZEN- O-16-glucoside, α-zearalenol, and α- zearalenol-sulfate. The nine biocontrol agents were divided into three groups: (1) *Rhizhopus oryzae* DSM 907 and *Rhizhopus stolonifera* DSM 855, which can catalyze ZEN glycosylation; (2) *Rhizhopus oryzae* DSM 906 and *Rhizhopus oligosporus* DSM 1964 and *Aspergillus oryzae* DSM 1864 and *Aspergillus oryzae* NBRC 100959, which are involved in the formation of sulfated ZEN metabolites; (3) Rhizopus DSM 908, DSM 1834, and Rhizopus oligosporus LMH 1133 T, which have shown the ability to produce the metabolite of both patterns [154]. The bacterial gut flora of pigs are able to transform ZEN into α-zearalenol and an unidentified compound via hydrolysis and DON into de-epoxy-DON via a de-epoxydation reaction [155]. 

OTA biodegradation by *Pediococcus parvulus* UTAD depended on the inoculum size and the incubation temperature coupled with a latency phase before biodegradation initiation. This later effect is due to the biodegradation enzyme synthesis of the bacteria [101]. OTA has been biodegraded into Otα by OTA amide group hydrolysis. On the other hand, OTA reduction by *Debaryomyces hansenii* involves neither absorption nor detoxification. It is a repression of the expression of the non-ribosomal peptide synthetase (otanpsPN) gene linked to the OTA biosynthetic pathway, which was observed in [48]. 

**Figure 2:**
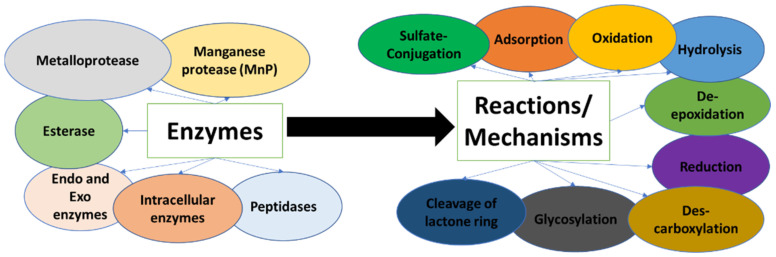
Mycotoxin biodegradation: Enzymes and reactions/mechanisms.

Generally, mechanisms of mycotoxin degradation by microorganisms include different types of enzymes (protease, esterase, intracellular enzymes, etc.). The degradation process can include one or two types of reactions. The mechanisms elucidated by now include oxidation, hydrolysis, the cleavage of the lactone ring, des-carboxylation, de-epoxidation, glycosylation, and sulfate-conjugation reactions. Figure 2 provides a general scheme of different enzymes that participate in mycotoxin degradation caused by microorganisms and the involved reactions.

Many studies have focused on the mycotoxin detoxification abilities of microorganisms, but a better understanding of responsible enzymes and the mechanisms involved is still needed. In some specific cases, no resulting metabolites were detected after mycotoxin biodegradation caused by microorganisms, but mostly, one or multiple compounds are usually detected. Table 6 provides an overview of mycotoxin degradation caused by microorganisms with a focus on the involved enzymes, degradation reactions, and resulting metabolites.

### 4.2. Decontamination by Removal Mechanism

The use of microorganisms as agents for toxin sequestration in order to remove them from food and feed is an approach that has shown many good results.

Taheur et al. showed that strains isolated from a kefir culture are efficient in binding mycotoxins. The binding ability was dependent on the strain and the mycotoxin type [158]. From the same perspective, *Saccharomyces cerevisiae CECT 1891* and *Lactobacillus acidophilus 24* FB1 were shown to have a binding ability by Pizzolitto et al. The binding process needed a little time (1 min), and the mechanism involved was demonstrated to be a toxin molecule via the physical adsorption of the microorganism’s cell wall components. Cell viability was not necessary for FB1 binding, but the microorganism’s cell wall structural integrity was required, and the process did not involve FB1 chemical modification [133]. From the same perspective, two strains of *Enterococcus faecium*, which are present in dairy products, particularly in cheese, are efficient in AFB1 and PAT removal [110]. The same results were obtained by Elsanhoty et al. when they studied the AFM1 removal ability of some strains of *Lactobacillus* in milk samples [159].

OTA removal by *Saccharomyces* strains was demonstrated by Bejaoui et al. to be an adsorption mechanism. This mechanism was dependent on the OTA molecule’s ionic properties, the yeast membrane state, and the biomass concentration [129].

*Lactococcus lactis* and *Bifidobacterium* sp. Isolated from milk are able to neutralize ZEN contents via absorption. The *Lactococcus lactis* absorption is not homogeneous, and the process happens in two different steps. The first one includes a ZEN absorption of 88%, and the second one consists of ZEN diffusion into bacterial cells. This was contrary to that of *Bifidobacterium* sp., where the adsorption mechanism only had a single homogeneous step. The deprotonated carboxyl groups of the bacterial proteins and peptidoglycan play a significant role in the absorption process [71].

AFB1 binding via the *Saccharomyces cerevisiae* mannoprotein is possible because of AFB1 absorption onto mannose sites, where the new structure is maintained. Indeed, the new structure nature does not match that of a natural AFB1 molecule, so AFB1 can be removed from the media [160].

### 4.3. Degradation Compound Toxicity

Knowing the degraded compound’s toxicity is very important because it can be more or less toxic than the parent. Therefore, many cytotoxicity studies have been conducted.

Adebo et al. studied the toxicity of the compounds resulting from AFB1 degradation caused by *Staphylococcus warneri*, *Sporosarcina* sp., and *Lysinibacillus fusiformis*. The experiment was conducted by monitoring the mortality of lymphocyte cells (from human blood) after the cells were exposed to degraded compounds. A lower mortality rate was recorded compared with aflatoxin B1. The authors concluded that there was lower toxicity [117]. On the other hand, *Escherichia coli* DH5a, *Arabidopsis thaliana*, and human hepatocyte LO2 were used by [138] to determine the degradation toxicity of the compound identified as desoxypatulinic acid (DPA) due to patulin detoxification caused by *Rhodosporidium paludigenum*. The lower toxicity of DPA compared with PAT was demonstrated.

Elsewhere, no toxicity reduction has been found after ZEN and FB1 biocontrol using lactic acid bacteria. One toxicity study was conducted using human esophageal carcinoma cell lines [124]. Some ZEN degradation products are known to be more toxic than ZEN. In the case of α-ZOL, it shows higher estrogenicity than ZEN [71]. The compounds derived from ZEN biocontrol toxicity can be ranked as follows: α-zearalenol > α-zearalanol > zearalenone > β-zearalenol [161].

**Figure 3:**
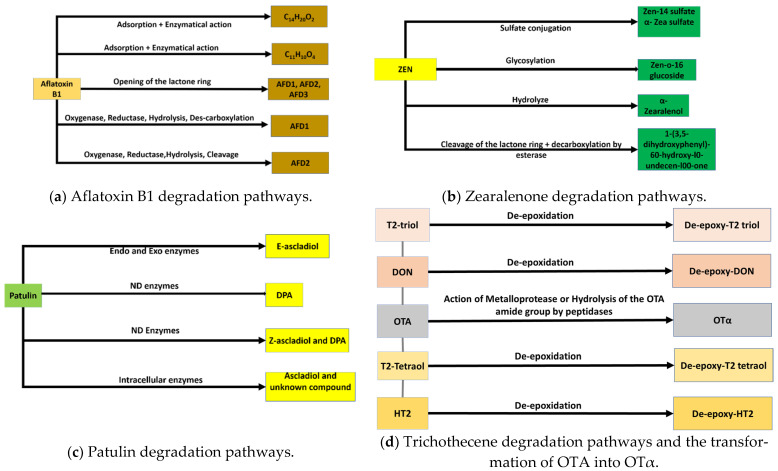
Some mycotoxin degradation pathways.

Figure 3 provides some mycotoxin degradation pathways.

## 5. Functional Enzymes Extraction from Bacteria

Nowadays, enzymes, as shown in Section 3.3, play a key role in mycotoxin biodegradation. Therefore, it is important to have a general method of enzyme extraction from microorganisms.

The process of enzyme extraction from microorganisms can be divided into three parts: extraction, purification, and characterization.

The extraction step’s main idea is to extract the enzyme outside the host. Some procedures are performed by harvesting the mycelia pellet via centrifugation and then washing it with phosphate buffer, followed by a second centrifugation to remove cell debris [127]. More recently, the homogenization of cells with protein extraction buffer followed by ultrasonication and centrifugation has been performed [148].

The purification step’s aim is, after the extraction step, to obtain an enzyme that is as pure as possible. Ammonium sulfate is the most used compound to precipitate enzymes [162]. This step is generally followed by centrifugation. In some cases, the precipitation step can be performed by using both organic solvents, such as methanol, ethanol, or acetone, and ammonium sulfate separately [163]. After enzyme activity determination, some purification techniques are used. Chromatography purification can be performed by using hydrophobic interaction chromatography (HIC) followed by immobilized metal ion affinity chromatography (IMAC) [127] or ion-exchange chromatography on a DEAE-Sepharose GE column, followed by dialysis and lyophilization [163]; dialysis can also be performed with a DEAE-Sepharose column [164]. Further purification can be performed using a Superdex 75 column followed by dialysis and lyophilization [163].

The last step is purified enzyme characterization. This step permits us to find the characteristics of the enzyme. It can be feasible to use SDS polyacrylamide gel electrophoresis (SDS-PAGE) to determine the molecular weight [127,163], HPTLC analysis to determine the enzymatic activity, and ESI-MS/MS to identify the enzyme [127]. Finally, the determination of the optimum pH, the optimum temperature, the ion metal effect on the enzyme activity [163], and the protein concentration (which can be determined using the method of Bradford) can be performed. Then, the enzyme can be stored at −85 °C until used.

**Figure 4:**
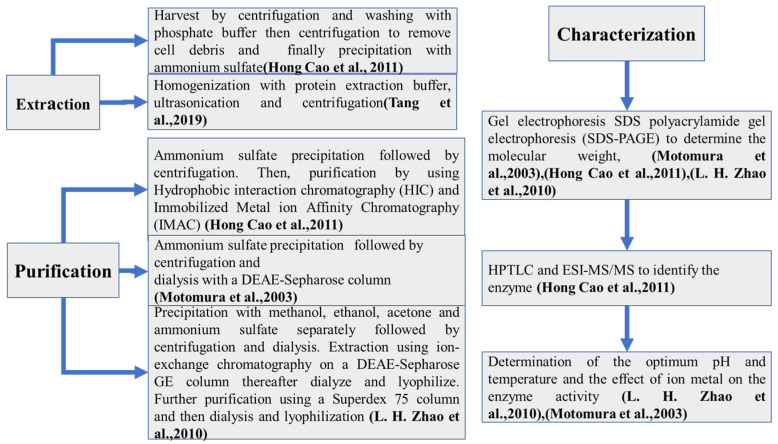
Functional enzyme extraction from bacteria. [127,163,164,165].

Figure 4 provides a brief scheme of all the steps of enzyme extraction from microorganisms.

## 6. Application and Perspectives

Microorganisms that can detoxify hazardous mycotoxins into low-toxicity compounds are of great importance. Being able to utilize them in the field and industries would be of great interest to food/feed safety. Therefore, it is advantageous to use microorganisms for mycotoxin detoxification on a large scale. Nevertheless, any applications to be set up must take into account both biocontrol agents and the life cycle of mycotoxigenic species, as well as the environmental conditions and plant agronomy [166]. Microorganisms that show activity against mycotoxins provide important properties because of the future possibility of exchanging the chemical and physical methods of preservation with a biological method based on those microorganisms and enzymes. Metabolism products of biocontrol agents are propitious for the bioconservation of food due to their ability to reduce the proliferation of mycotoxigenic fungi and mycotoxin production [167].

Microorganisms are used in many different ways. They are already used as probiotics to enhance the health of the host upon adequate administration. *Lactobacillus* species are most often used as probiotics [168], mainly via encapsulation [169], [170]. Encapsulation is one of the most effective methods of saving the viability and stability of microorganisms [113,171]. Therefore, it is a good alternative for microorganism applications in food and feed. Recently, the yeast *Sporidiobolus pararoseus*, which has a mycotoxin binding ability, was successfully produced with this approach on an industrial production scale with possible applications in feed additives [172].

Microorganisms can also be used as biopesticides [173]. The use of biofungicides is an approach that involves the application of different microorganisms that can suppress toxic fungi [174]. Recently, novel biofungicide formulations based on *Bacillus subtilis* 5, *Bacillus cereus* 3S5, and *Pseudomonas fluorecens* 10S2 were produced [175]. The same formulation has been created using other microorganisms [176,177,178].

Finally, the mycotoxin degradation enzyme can be especially valuable in the feed, food, and fermentation industry [109,120]. The α-amylase enzymes from some bacterial or fungal strains are widely used [179]. A carboxypeptidase that can degrade OTA has been cloned and used to detoxify the OTA mycotoxin [145].

## 7. Conclusions

This paper reviews mycotoxin degradation caused by microorganisms. Mycotoxins are secondary compounds produced by fungi with various chemical structures. Some of them are very hazardous for humans and animals, and strict regulations have been made for their content in food and feed. Physical, chemical, and biological methods can be used to control mycotoxin food/feed contamination. Biological control, which includes bacteria, yeast, and enzymatic activities against mycotoxins, is considered a very friendly control method compared with physical and chemical methods. However, more studies are needed to elucidate mycotoxin detoxification mechanisms. Unfortunately, most investigations do not address the real process involved with this biodegradation. In some cases, the degradation compound structures are elucidated, which helps to provide the hypothetically involved mechanisms. The detoxification of mycotoxins using bacterial strains augurs a new path for food/feed safety. From this perspective, more emphasis can be given to the toxicity of the resulting degradation compounds and the involved mechanisms of elucidation.

## Figures and Tables

**Table 1:** Some mycotoxin characterizations.

Toxins	Effects	Fungi Producer	WHO Recommendation	References
Aflatoxin B1	Cancerogenic, teratogenic, mutagenic	*Aspergillus flavus*, *Aspergillus parasiticus*, *Aspergillus nominus*, and *Aspergillus niger*	15 µg/kg in peanuts	[5,30,31,39,78,79,80]
Patulin	Genotoxicity, mutagenicity, gastrointestinal disorders, edema	*Penicillium*, *Byssochlamys*, and *Aspergillus* species	50 µg/kg in apple juice	[27,81,82,83,84,85]
OTA	Nephrotoxic and neurotoxic effects, affects mammary functions	*Aspergillus ochraceus*, *Penicillium verrucosum*, *Aspergillus carbonarius*, and *Aspergillus niger*	5 µg/kg in wheat and barley	[50,52,54,86,87]
DON	Intestinal damage, emetic effects, immune-toxic	*Fusarium graminearum* and *Fusarium culmorum*	2000 µg/kg in wheat, barley, and maize	[88,89,90]
ZEN	Cytogenetic toxicity, decreases fertility, embryotoxicity, immunotoxicity, estrogenic, anti-androgenic activities	*Fusarium graminearum*, *Fusarium culmorum*, *Fusarium cerealis*, *Fusarium equiseti*, and *Fusarium semitectum*	TDI^1^ 0.25 µg/kg by EFSA^2^	[70,71,72,73,74,91,92,93]
Fumonisin B1	Neurotoxicity, Hepatotoxicity, nephrotoxicity	*Fusarium verticilloides* and *Fusarium proliferatum*	Total of FB_1_ + FB_2_: 2000 µg/kg in maize flour and maize meal	[59,94,95,96]

^1^TDI: Tolerable daily intake; ^2^EFSA: European Food Safety Authority.

**Table 2:** Aflatoxin B1 detoxification by bacteria.

Bacteria	Medium Culture	Main Effects	References
*Bacillus subtilis* UTBSP1	(1) Nutrient broth culture (2) Pistachio nut	Detoxification of AFB1 by 85.66% and 95%, respectively, in the nutrient broth culture and the pistachio nuts in optima conditions of 35–40 °C during 24 h.	[109]
*Mycobacterium fluoranthenivorans* sp.	Medium culture with AFB1	The AFB1 concentration was reduced by 70% to 80% within 36 h.	[115]
*Myroides odoratimimus* strain 3J2MO	Medium culture with AFB1	Degradation of 93.82% of the AFB1 after incubation for 48 h at 37 °C.	[116]
*Pseudomonas fluorescens* strain 3JW1	(1) Medium culture with AFB1 (2) Peanut medium (3) Peanut kernels	Degradation of AFB1 by 88.3% in 96 h.	[18]
*Rhodococcus pyridinivorans* K408	Bioethanol produced by *Aspergillus flavus*-contaminated corn	Degradation rate was more than 63% in the solid phase and 75% in the liquid phase after 12 experiment days.	[26]
*Staphylococcus warneri, Sporosarcina* sp., *Lysinibacillus fusiformis*	Medium culture with AFB1 standard	Both cultures and lysates degraded AFB1, and the addition of a protease inhibitor enhanced the degradation rate of the lysate.	[117]
*Enterococcus faecium* M74 and EF031 strains	Medium culture with FB1 solution	AFB1 removal by 19.3 to 30.5% for M74 strain and 23.4 to 37.5% for EF031 strain.	[110]
*Pontibacter* specie	Medium culture with aflatoxin B1 standard	Lysates and cultures both degraded AFB1.	[118]
Microbial consortium, *TADC7*	Medium culture with aflatoxin B1 standard	Degradation of more than 95% of the amount of AFB1 after five days cultivation in PCS medium at 55 °C.	[107]
*Lacticaseibacillus rhamnosus* (previously *Lactobacillus rhamnosus)* strains LBGG and LC705	Medium culture with aflatoxin B1 standard	A rapid removal of 80% of AFB1 by both two strains.	[108]
*Lacticaseibacillus rhamnosus* (previously *Lactobacillus rhamnosus*) *TISTR 541*	Bread produced by contaminated wheat flour	Decrease in AFB1 levels during mixing and fermentation process.	[119]
*Rhodococcus erythropolis*	Medium culture with aflatoxin B1 standard	A significant reduction in the amount of AFB1 when treated with the *Rhodococcus erythropolis* extracellular extracts.	[120]
*Lactobacillus acidophilus and prebiotics*	Whole cow’s milk	Reduction in AFB1 of 13.53 to 35.53%.	[103]
*Lactobacillus acidophilus and Lacticaseibacillus* (previously *Lactobacillus rhamnosus*)	Yogurt samples	Binding of AFB1 by 64.56 to 96.58% during 21 days of storage.	[121]

**Table 3:** Other mycotoxins detoxification by bacteria.

Bacteria	Toxins	Medium Culture	Main Effects	References
*Enterococcus faecium* M74 and EF031 strains	Patulin	Medium culture enriched with patulin solution	Patulin removal of 15.8 to 41.6% for M74 strain and 19.5 to 45.3% for EF031 strain.	[110]
*Bacillus pumilus ES-21*	Zearalenone	Medium culture with ZEN standard	The degradation rate was more than 95.7%.	[91]
*Bacillus amyloliquefaciens* ZDS-1	Zearalenone	(1) Medium culture with ZEN standard (2) Contaminated wheat samples	ZEN degradation with a concentration ranging from 1 mg/L to 100 mg/L for specific optimal conditions, which are temperature 30 °C, pH from 6.0 to 7.0, and a microorganism concentration of 5.1 × 10^8^ CFU/mL.	[122]
*Rhodococcus pyridinivorans* strains (K408 and AK37)	AFB1, T2 toxin, ZEA	Medium culture with mycotoxin standard solutions	Degradation of the 03 mycotoxins and increase in the ZON degradation capacity from 60% to 95% in the multi-mycotoxin degradation system	[22]
Microbial consortium TADC7	AFB1, ZEN	Medium culture with mycotoxin standard solutions	Degradation of AFB1 by 98.9% and ZEN by 88.5% after 168 h.	[111]
*Pseudomonas otitidis* TH-N1	Zearalenone	Medium culture with a ZEN standard	Degradation of ZEN under optimal conditions: Temperature 37 °C, pH 4 to pH 5, and bacterial concentration of 10^9^ CFU/mL.	[105]
Bacterial consortium PGC-3	DON, NIV	Medium culture with mycotoxin standard solution	Biotransformation of DON into de-epoxy-DON and NIV into de-epoxy-NIV with optimal conditions of pH 5–10 and temperatures of 20–37 °C in aerobic conditions.	[123]
*Lactic acid bacteria*	DON, T-2, HT-2, ZEN	Malting wheat	Reduction in the amount of DON, T-2, HT-2, and ZEN of, respectively, 23%, 34%, 58%, and 73% in malting wheat samples.	[112]
*Lactic acid bacteria*	FB1, ZEN	Maize meal	Reduction in ZEN of 68.3% and FB1 of 75% after 4 incubation days.	[124]
*Limosilactobacillus reuteri* (previously *Lactobacillus reuteri*)	ZEN	Nutrient broth and maize kernels	Hydrolysis of 5.0 mg/L ZEN for 8 h in nutrient broth and hydrolysis of 2.5 mg/kg ZEN for 4 h in ZEN-contaminated maize kernels.	[125]
*Bacillus velezensis Strain ANSB01E*	ZEN	Liquid medium and moldy corn	ZEN degradation of 95% in the liquid medium and of 25% in the moldy corn after 48 h.	[106]

**Table 4:** Toxin detoxification by yeasts.

Yeasts	Toxins	Medium Culture	Main Effects	References
*Saccharomyces cerevisiae*	Beauvericin (BEA)	(1) Standard of BEA (2) Corn flour	In total, 89.1 to 99.3% degradation rate in the standard solution against 73.5 to 91% in the cornflour.	[137]
*Rhodosporidium paludigenum*	Patulin	Patulin standard	Removal of the total amount of patulin after two days at 28 °C.	[138]
*Armillariella tabescens*	Aflatoxin B1	Aflatoxin B1 standard	Cleavage of the bis-furan ring.	[127]
*Candida versatilis CGMCC 3790*	Aflatoxin B1	A mixture of steamed soybean and baked wheat flour	Degradation dependent on initial AFB1 concentration.	[139]
*Rhizopus stolonifer*	OTA	Wheat contaminated by OTA	Degradation of 96.5% of OTA.	[140]
*Candida intermedia, Lachancea thermotolerans, Candida friedrichii*	OTA	Grape juice	Reductions in OTA by *Candida intermedia, Lachancea thermotolerans, Candida friedrichii* of 73%, 75%, and 70%, respectively.	[136]
*Candida tropicalis, Torulaspora delbriickii, Zygosaccharomyces rouxii*, and *Saccharomyces* strains	ZEN	Growth media	Biodegradation of ZEN into α- zearalenol and β-zearalenol.	[141]
*Saccharomyces cerevisiae W13*	OTA	Semi-synthetic medium	Removal of an amount of OTA from 6 to 57.21% with the highest level obtained at 30 °C with 250 g/L of sugar.	[130]

**Table 5:** Toxin detoxification by enzymes.

Toxins	Medium	Enzymes	Main Effects	References
Patulin	Apple juice	Orotate phosphoribosyltransferase	The degradation rate can reach over 80%.	[148]
Aflatoxin B1	Medium culture with aflatoxin B1 standard	Aflatoxin-oxidase (AFO)	Cleavage of the bis-furan ring.	[127]
Aflatoxin B1	Citrate buffer solution containing 20% DMSO	Laccase	Under optimal conditions, which are a temperature of 35 °C, a pH of 4.5, and a laccase activity of 30 U/mL, 67% of the AFB1 total amount was degraded after two days.	[144]
OTA	LB medium	Carboxypeptidase from *Bacillus amyloliquefaciens* ASAG1	Decrease of 41% and 72%, respectively, when cultivated with the supernatant and the purified protein of carboxypeptidase.	[145]
OTA	Buffer systems with enzymes	Commercial protease A, commercial pancreatin, and an enzyme extract isolated from *Aspergillus niger* MUM	At pH 7.5 and 37 °C, protease A and pancreatin reduce the OTA level, respectively, by 87.3%, 43.4%, and 99.8% after 25 h.	[146]
AFB1	Reaction mixture	Manganese protease MnP	In total, 86% of AFB1 levels decrease after 48 h and 5 nkat of MnP.	[143]
Patulin	Pear juice	Porcine pancreatic lipase (PPL)	Patulin degradation with 0.02 g/mL PPL and 0.375 mg/L of PAT at 40 °C within 24 h.	[147]
Aflatoxin B1, Fumonisin B1, Ochratoxin A, Zearalenone, T-2	Medium culture with a standard solution of mycotoxins	Ery4 laccase from *Saccharomyces cerevisiae*	AFB1, FB1, OTA, ZEN, and T-2 toxin degradations of 73%, 74%, 27%, 100%, and 40%, respectively.	[142]
Aflatoxin B1	Medium culture with aflatoxin B1 standard	Laccase from white rot fungi	In total, 40.45% degradation of AFB1 by *Peniophora* sp. SCC0152; 35.90% degradation of AFB1 by *Pleurotus ostreatus* St2; 3; 87.34% degradation of AFB1 by pure laccase from *Trametes versicolor*.	[149]

**Table 6:** Toxin degradation by microorganism mechanisms summary.

Microorganisms	Genes or Enzymes	Toxins	Degradation Reactions	Obtained Metabolites	References
*Tetragenococcus halophilus*	Enzyme ND *	Aflatoxin B1	Adsorption + enzymatical action	C_14_H_10_O_4_, C_18_H_16_O_8_, C_14_H_12_O_3_, C_16_H_20_O_4_, C_14_H_16_O_2_, C_14_H_20_O_2_	[99]
*Candida versatilis* CGMCC 3790	Enzyme ND	Aflatoxin B1	Adsorption + enzymatical action	C_14_H_10_O_4_, C_14_H_12_O_3_, C_13_H_12_O_2,_ C_11_H_10_O_4_.	[139]
*PhanerochaetesordidaYK-624*	Manganese protease MnP	Aflatoxin B1	Oxidation + hydrolysis	AFB1-8,9-dihydrodiol	[143]
*Rhodosporidium paludigenum*	Enzyme ND	Patulin	ND	Desoxypatulinic acid	[138]
*Bacillus pumilus ES-21*	Esterase	Zearalenone (ZEN)	Cleavage of the lactone ring, followed by des-carboxylation. The enzymatic process follows first-order kinetics with t_1/2_ of 6.52 h.	1-(3,5-dihydroxyphenyl)-60-hydroxy-l0- undecen-l00-one	[91]
*Eggerthella* sp.	Enzyme ND	DON, HT-2, T-2 triol and T-2 tetraol	De-epoxidation	De-epoxy- DON, de-epoxy T-2triol, de-epoxy HT-2, de-epoxy T-2 tetraol for the 04 parents in different ratios	[62]
*Saccharomyces cerevisiae*	Endo and Exo enzymes synthesized by the yeast during the fermentation	Patulin	The mechanism was enzymatical and the production of the relevant PAT-metabolizing enzymes synthesized by the yeast cells is not induced by PAT preincubation	E-ascladiol	[27]
*Sporobolomyces* sp. IAM 13481	ND	Patulin	The mechanism was induced by pretreatment with patulin.	DPA and (Z)-ascladiol	[152]
*Pediococcus parvulus* UTAD	Peptidases	OTA	Hydrolysis of the OTA amide group.	Otα	[101]
*Rhizopus* and *Aspergillus* species		ZEN	Glycosylation, sulfate-conjugation	ZEN-14-sulfate, ZEN-O-14, ZEN- O-16-glucoside, α-zearalenol, α- zearalenol-sulfate	[154]
*Phaffia rhodozyma*	Metalloprotease	OTA	ND	Otα	[156]
*Gut Microflora of Pigs*	ND	DON	De-epoxidation	De-epoxy-DON	[155]
*Gut Microflora of Pigs*	ND	ZEN	Hydrolyze	α-zearalenol	[155]
*Bacillus subtilis UTB1*	Gene bacC,	AFB1	Reduction in the double bond Hydrolysis of the ester bond Des-carboxylation	AFD1	[150]
*Pseudomonas putida*	ND	AFB1	Opening of the lactone ring	AFD1, AFD2, AFD3	[151]
*Pichia caribbica*	Intracellular enzymes	Patulin	Unidentified	Ascladiol and unknown compound	[157]

* ND: not determined.

## Data Availability

Not applicable.
